# Monitoring Intra-cellular Tacrolimus Concentrations in Solid Organ Transplantation: Use of Peripheral Blood Mononuclear Cells and Graft Biopsy Tissue

**DOI:** 10.3389/fphar.2021.733285

**Published:** 2021-10-26

**Authors:** Benedetta C. Sallustio

**Affiliations:** ^1^ Department of Clinical Pharmacology, Basil Hetzel Institute for Translational Health Research, The Queen Elizabeth Hospital, Woodville South, SA, Australia; ^2^ Discipline of Pharmacology, School of Medicine, University of Adelaide, Adelaide, SA, Australia

**Keywords:** tacrolimus, transplantatation, rejection, nephrotoxicity, therapeutic drug monitoring, intra-cellular concentrations

## Abstract

Tacrolimus is an essential immunosuppressant for the prevention of rejection in solid organ transplantation. Its low therapeutic index and high pharmacokinetic variability necessitates therapeutic drug monitoring (TDM) to individualise dose. However, rejection and toxicity still occur in transplant recipients with blood tacrolimus trough concentrations (C_0_) within the target ranges. Peripheral blood mononuclear cells (PBMC) have been investigated as surrogates for tacrolimus’s site of action (lymphocytes) and measuring allograft tacrolimus concentrations has also been explored for predicting rejection or nephrotoxicity. There are relatively weak correlations between blood and PBMC or graft tacrolimus concentrations. Haematocrit is the only consistent significant (albeit weak) determinant of tacrolimus distribution between blood and PBMC in both liver and renal transplant recipients. In contrast, the role of *ABCB1* pharmacogenetics is contradictory. With respect to distribution into allograft tissue, studies report no, or poor, correlations between blood and graft tacrolimus concentrations. Two studies observed no effect of donor *ABCB1* or *CYP3A5* pharmacogenetics on the relationship between blood and renal graft tacrolimus concentrations and only one group has reported an association between donor *ABCB1* polymorphisms and hepatic graft tacrolimus concentrations. Several studies describe significant correlations between *in vivo* PBMC tacrolimus concentrations and *ex vivo* T-cell activation or calcineurin activity. Older studies provide evidence of a strong predictive value of PBMC C_0_ and allograft tacrolimus C_0_ (but not blood C_0_) with respect to rejection in liver transplant recipients administered tacrolimus with/without a steroid. However, these results have not been independently replicated in liver or other transplants using current triple maintenance immunosuppression. Only one study has reported a possible association between renal graft tacrolimus concentrations and acute tacrolimus nephrotoxicity. Thus, well-designed and powered prospective clinical studies are still required to determine whether measuring tacrolimus PBMC or graft concentrations offers a significant benefit compared to current TDM.

## Introduction

The first calcineurin inhibitor (CNI), ciclosporin, revolutionised solid organ transplantation in the early 1980s providing, for the first time, immunosuppression that selectively targeted T-cell mediated rejection. Calcineurin is a serine-threonine phosphatase that dephosphorylates nuclear factor of activated T-cells (NFAT) allowing the translocation of this nuclear receptor into the nucleus, initiating T-cell activation *via* upregulation of interleukin-2 expression (Brunet et al., 2019). The second CNI, tacrolimus entered clinical use in the early 1990s, further reducing the incidence of rejection, and rapidly becoming the cornerstone of maintenance immunosuppression in solid organ transplantation. Tacrolimus inhibits calcineurin by binding to its cytosolic receptor, FKBP12. Both ciclosporin and tacrolimus have low therapeutic indices and, due to their central CNI mechanism of action, have overlapping spectra of adverse effects including nephrotoxicity, one of the major dose-limiting toxicities (Brunet et al., 2019).

Tacrolimus pharmacokinetics display significant inter-individual variability due to differences in the hepatic and intestinal expression/activity of cytochrome P450 (CYP) 3A4, CYP3A5 and P-glycoprotein, arising from the effects of genetic polymorphisms, drug- and environmental-interactions (Christians et al., 2002; Staatz and Tett, 2004; Brunet et al., 2019). The latter two also contributing to intra-individual pharmacokinetic variability. Pharmacokinetic variability together with tacrolimu’s low therapeutic index has led to dosage individualisation using therapeutic drug monitoring (TDM) (Staatz and Tett, 2004). During its early clinical use blood tacrolimus concentration-response relationships with respect to rejection and/or adverse effects were described in clinical studies of renal and hepatic transplant recipients administered tacrolimus alone or with a steroid (with or without azathioprine) (Kershner and Fitzsimmons, 1996). However, despite relatively high therapeutic ranges, a continuing significant incidence of rejection and nephrotoxicity spurred the development of induction therapy and the establishment of triple maintenance immunosuppression consisting primarily of tacrolimus co-administered with a corticosteroid and mycophenolic acid (Wallemacq et al., 2009). This has allowed a significant decrease in the targeted therapeutic range of whole blood trough tacrolimus concentrations (C_0Blood_), minimising long-term nephrotoxicity (Brunet et al., 2019) whilst still maintaining an acceptably low risk of rejection. However, the effectiveness of the therapeutic range is controversial, and rejection still occurs in patients with C_0Blood_ within the current therapeutic ranges (Wallemacq et al., 2009; Hu et al., 2019).

**FIGURE 1:**
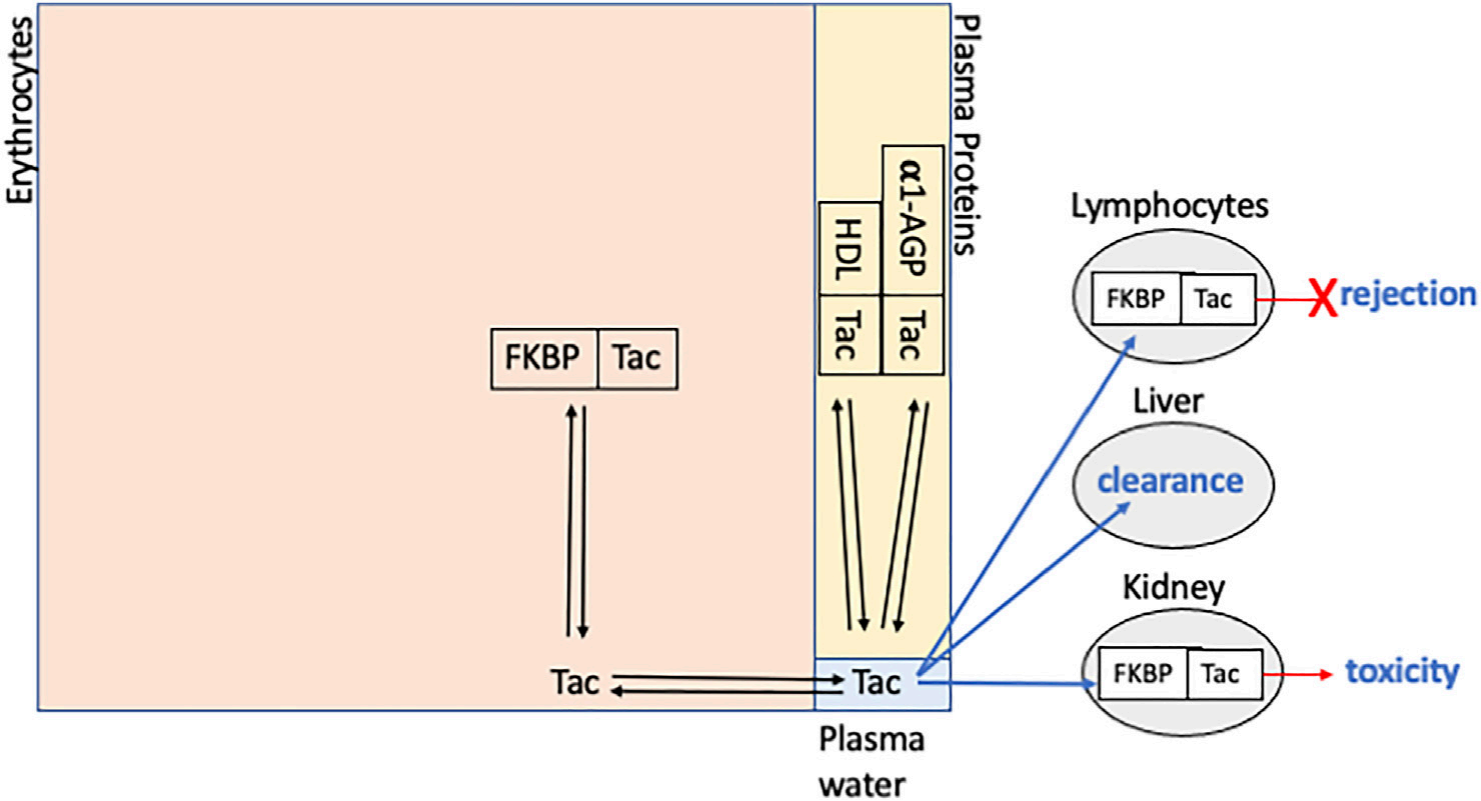
Schematic representation of tacrolimus (Tac) distribution within whole blood showing binding to FKBP12 (FKBP), high density lipoproteins (HDL) and alpha-1 acid glycoprotein (α1-AGP). The large rectangle is divided into sections whose area approximates relative distribution between erythrocytes, plasma proteins and plasma water (unbound tacrolimus). The PBMC compartment is not shown and represents < 1% of tacrolimus in whole blood. Blue arrows indicate distribution into: lymphocytes, where biding with FKBP12 results in inhibition of calcineurin and prevention of rejection; liver, the primary site of clearance; and kidneys, where binding with FKBP12 and inhibition of calcineurin may be associated with nephrotoxicity.

In whole blood tacrolimus distributes primarily within erythrocytes (approximately 85%), another 14% is distributed in plasma and only a small proportion (< 1%) is in the mononuclear cell fraction that contains lymphocytes (Figure 1) (Zahir et al., 2001; Zahir et al., 2004a; Zahir et al., 2004b). In plasma tacrolimus binds to soluble proteins and, to a lesser extent, lipoproteins, resulting in a low plasma unbound fraction (approximately 1%) (Zahir et al., 2001; Zahir et al., 2004a; Zahir et al., 2004b). Haematocrit and erythrocyte numbers significantly affect the distribution of tacrolimus into plasma (Zahir et al., 2004a), as does concentration-dependent binding within erythrocytes (Zahir et al., 2001). Although plasma protein binding is not concentration-dependent, it is significantly affected by plasma α1-acid glycoprotein and HDL-cholesterol concentrations (Zahir et al., 2004a). Since only unbound tacrolimus is available for distribution into lymphocytes and other tissues where it exerts pharmacological activity, the use of blood concentrations for tacrolimus TDM is problematic as changes or differences in blood concentrations may reflect alterations in binding to erythrocytes or plasma proteins, rather than any differences in unbound tacrolimus concentrations. This has led to considerable effort to measure tacrolimus concentrations directly at its sites of immunosuppression (lymphocytes) and of toxicity. Peripheral blood mononuclear cells (PBMC) are a readily accessible matrix that has been investigated as a convenient surrogate for lymphocyte tacrolimus concentrations. Allograft tissue biopsy samples have similarly been investigated as a potential adjunct to tacrolimus TDM. In 2016 Capron et al. (2016) reviewed the potential of monitoring intra-cellular immunosuppressant drug concentrations in transplantation, and Lemaitre et al. (2020) recently published an expert consensus on requirements for measuring PBMC tacrolimus concentrations. This review will update the evidence for tacrolimus concentration-effect relationships using either PBMC or graft tissue; the pharmacokinetics of tacrolimus in these biological matrices; and the relationship between blood, PBMC and graft tacrolimus concentrations.

## PBMC Tacrolimus Concentrations

### Rejection and Nephrotoxicity

Zahir *et al.* provided the first evidence for a possible clinical benefit of measuring PBMC tacrolimus concentrations in a study of 40 adult liver transplant recipients. They reported that a lower proportion of total blood tacrolimus was associated with the leucocyte fraction in patients with rejection compared to those without (Zahir et al., 2004a; Zahir et al., 2004b). In 90 adult liver transplant recipients studied 7 days post transplantation, Capron et al. (2012) later observed that whilst there was no relationship between C_0Blood_ and rejection, trough PBMC tacrolimus concentrations (C_0PBMC_) were significantly lower in patients with rejection compared to those without, regardless of whether rejection was classified histologically or clinically (Table 1A). Importantly, C_0PBMC_ measured on days 3 and 5 were also significantly different between recipients who would be classified as rejectors or non-rejectors on day-7, suggesting the potential to predict early rejection, In addition, day-7 C_0PBMC_ correlated with the histological grading of rejection. To date, this is the only study that has directly and prospectively compared prediction of rejection by C_0Blood_ and matched C_0PBMC_ taken on the same day as the protocol liver biopsy used to classify rejection. However, patients received only tacrolimus monotherapy for maintenance immunosuppression, with or without anti-lymphocytic induction therapy, and there was a high incidence (41%) of moderate/severe histological rejection.

**TABLE 1:** Summary of clinical studies investigating blood, PBMC and allograft tacrolimus concentrations and their associations with clinical outcomes or *ex-vivo* pharmacodynamic assessments.

Study transplant type	Time post-transplant	Maintenance immuno-suppresion	Analytical methods and sample collection times	Interacting drugs	Main clinical outcomes or *ex vivo* pharmacodynamic outcomes
A. Clinical Outcomes					
Sandborn et al. (1995)Adult Liver (*n* = 17)	Up to 8 weeks	Tac, steroid	**Plasma** and **graft tissue**: IA (non-specific)C_0_	Not stated	Prospective observational studyBased on protocol and for-cause biopsies, liver [Tac] significantly lower in rejectors compared to non-rejectors. No difference in plasma [Tac]
Capron et al. (2007)Adult Liver (*n* = 146)	Day 7	Tac ± steroid	**Blood**: IA (non-specific) **graft tissue**: LC-MS/MSC_0_	Ceased by day 7	Prospective observational studyBased on day 7 protocol biopsies, liver [Tac] 30 pg/mg cut-off (sensitivity 89%, specificity 98%) predicts clinically significant rejection
Capron et al. (2012)Adult Liver (*n* = 90)	Day 7	Tac	**Blood**: IA**PBMC** and **graft tissue**: LC-MS/MSC_0_	Excluded	Prospective observational studyBased on day 7 protocol biopsies, significant association between severity of rejection and C_0PBMC_ or C_0Liver_. No relationship with C_0Blood_
Rayar et al. (2018)Adult Liver (*n* = 41)	Days 1–7	Tac, MPA, steroid	**Blood** and **PBMC**: LC-MS/MSC_0_	Not stated	Prospective observational studyNo significant independent associations of C_0PBMC_ with measures of graft function
Han et al. (2016)Adult Kidney (*n* = 214)	SS up to 14 years	Tac, MPA, steroid	**Blood** and **PBMC**: LC-MS/MSC_0_	Excluded	Prospective observational PK-*ex vivo* PD study. Retrospective analysis of rejection and tacrolimus-induced nephrotoxicity. No significant association between C_0PBMC_ and history of acute rejection or nephrotoxicity in first 6 months post-transplant
Francke et al. (2020)Adult Kidney (*n* = 175)	3, 6 and 12 months	Tac, MPA, steroid	**Blood**: IA**PBMC**: LC-MS/MSC_0_	Excluded up to 3 months. Unclear for >3 months	Prospective observational PK study. Retrospective analysis of rejection and tacrolimus-induced nephrotoxicity. Based on for-cause biopsies, no association between the 3-month C_0PBMC_ or C_0Blood_ and rejection within the first 3 months post-transplant. Similarly, no associations with clinically defined nephrotoxicity or new onset diabetes mellitus within the first 3 months post-transplant
Zhang et al. (2020)Adult Kidney (*n* = 52)	3 months and 1 year	Tac, MPA, steroid	**Blood**: IA **graft tissue**: LC-MS/MSC_0_	Not stated	Prospective observational studyBased on protocol biopsies, no association between renal [Tac] and subclinical acute rejection at either 3 months or 1 year
Sallustio et al. (2021)Adult Kidney (*n* = 132)	SS 15 (8–80)^7^ days	Tac, MPA, steroid	**Blood** and **graft tissue**: LC-MS/MSC_0_	Not excluded	Prospective observational studyBased on protocol and for-cause biopsies, no association between renal [Tac] and rejection. C_0Blood_, dose and acute nephrotoxicity were associated with renal [Tac]
B. *Ex-vivo* Pharmacodynamic Assessments					
Lemaitre et al. (2015)Adult Liver (*n* = 10)	Days 1 and 7	Tac, MPA, steroid	**Blood** and **PBMC**: LC-MS/MSC_max_, C_12_ and AUC	Anti-retrovirals excluded	Prospective observational studyOn day 1 changes in CNA mirrored those in blood and PBMC [Tac]. No correlations between AUC_CNA_ and either AUC_Blood_ or AUC_PBMC_.
Tron et al. (2020)Adult Liver (*n* = 32)	SSDay 7–10	Tac, MPA, steroid	**Blood** and **PBMC**: LC-MS/MSC_0_, C_max_ and AUC	Excluded	Prospective observational studyNo correlation between AUC_CNA_ and either AUC_Blood_ or AUC_PBMC_. Significant association between maximal inhibition of CAN and either log AUC_PBMC_ or log AUC_Blood_
Han et al. (2016)Adult Kidney (*n* = 214)	SS up to 14 years	Tac, MPA, steroid	**Blood** and **PBMC**: LC-MS/MSC_0_	Excluded	Prospective observational studyIn sub-group (*n* = 39), both C_0PBMC_ and C_0Blood_ associated with *ex vivo* measures of T-cell activation
Fontova et al. (2021)Adult Kidney (*n* = 25)	SS > 6 months a.m. and p.m. dose	Tac, MPA, steroid	**Blood** and **PBMC**: LC-MS/MSC_max_, C_12_ and AUC	Excluded	Prospective observational studySignificant correlation between blood [Tac] and CNA over a 24 h (a.m. plus p.m.) dosing interval. Correlation between PBMC [Tac] and CNA not investigated

Tac = tacrolimus, [Tac] = tacrolimus concentration, MPA = mycophenolic acid, SS = steady state, CNA = calcineurin activity, PK = pharmacokinetic, PD = pharmacodynamic.

Two other clinical studies that measured PBMC tacrolimus concentrations in liver transplants have also included clinical outcome data. These more recent studies recruited patients receiving triple maintenance immunosuppression with tacrolimus, mycophenolic acid and a corticosteroid (Lemaitre et al., 2015; Rayar et al., 2018). In 10 transplant recipients recruited at steady state (Table 1B), only one case of acute rejection was observed precluding any investigation of the relationship between rejection and tacrolimus concentrations in PBMC (Lemaitre et al., 2015). A study of 41 patients (Table 1A) recruited in the first week post transplantation did not collect data on rejection but found no association between C_0PBMC_ and measures of graft function (Rayar et al., 2018).

Only three studies in kidney transplant recipients provide any data on clinical outcomes and tacrolimus concentrations in blood and PBMC (Francke et al., 2020; Han et al., 2016; Klaasen et al., 2018). Again, transplant recipients received triple maintenance immunosuppression as above. In a small study of 29 patients (Supplementary Table S1), the incidence of rejection 1 week post-transplant was too low to adequately investigate any relationship with PBMC tacrolimus concentrations (Klaasen et al., 2018). Two larger studies (Francke et al., 2020; Han et al., 2016) carried out at steady state assessed clinical outcomes retrospectively (Table 1A). Han *et al.* reported 15.6% of kidney transplant recipients experienced rejection within the first 6 months post-transplant (Han et al., 2016). However, the authors did not state how rejection was defined and found no association between rejection and C_0PBMC_ measured in 214 patients up to 14 years post-transplant. Using only for-cause biopsies in 175 recipients, Francke *et al.* reported 8% developed biopsy proven rejection in the first 3 months of renal transplantation, with no difference in the 3-month C_0PBMC_, C_0Blood_ or their ratio between patients who had and hadn’t experienced rejection (Francke et al., 2020). Both studies (Han et al., 2016; Francke et al., 2020) also investigated tacrolimus-induced nephrotoxicity and found no difference between C_0PBMC_ in patients who had or hadn’t experienced nephrotoxicity over the first 6 or 3 months of renal transplantation, respectively.

Overall, there is very little evidence for clinical utility of C_0PBMC_ in the prediction of rejection or tacrolimus-induced nephrotoxicity. This may mostly be due to small sample sizes, the low incidence of rejection with modern triple maintenance immunosuppression, and the often retrospective assessment of rejection and nephrotoxicity. The only clinical evidence for prediction of rejection is from a single well-designed study in liver transplant patients receiving tacrolimus monotherapy, which may not be directly translatable to patients on modern maintenance immunosuppression or to other transplant types.

### *Ex vivo* Calcineurin Activity and T-Cell Activation

In the absence of sufficient clinical outcome data, several groups have investigated the relationship between blood or PBMC tacrolimus concentrations and *ex vivo* calcineurin activity (CNA) or T-cell activation (Table 1B). In 10 *de novo* liver transplant recipients studied on days 1 and 7 following commencement of tacrolimus therapy Lemaitre et al. (2015) reported that, over a 12-h dosing interval, inhibition of PBMC CNA on day 1 mirrored tacrolimus concentrations in both blood and PBMC, with an average maximum inhibition of 38% occurring slightly after attainment of maximum tacrolimus concentrations in blood (C_maxBlood_) and PBMC (C_maxPBMC_). On day 7, no change in CNA was observed over the dosing interval, and less peak to trough variability in the tacrolimus concentration *vs* time curves was apparent in both blood and PBMC compared to day 1. There was no correlation between the 12-h area under the CNA *vs* time curve (AUC_CNA_) on days 1 or 7 and the corresponding 0–12 h areas under the tacrolimus concentration *vs* time curves in whole blood (AUC_Blood_) or PBMC (AUC_PBMC_). In this study, although PBMC tacrolimus concentrations and CNA were measured in the whole PBMC fraction, the authors accounted for granulocyte contamination of PBMC by expressing the tacrolimus concentrations per 10^6^ leukocytes, rather than the conventional use of total cells. However, tacrolimus would likely also distribute into granulocytes, potentially biasing the relationship between measured intracellular tacrolimus concentrations and CNA.

In 32 *de novo* liver transplant recipients studied between day 7–10 post-transplant, Tron et al. (2020) confirmed that maximal inhibition of CNA (CNA_Imax_) occurred 2 h post-dose, slightly after attainment of C_maxBlood_ and C_maxPBMC_ (1.6 h post-dose). CNA_Imax_ was correlated with both log-C_maxPBMC_ and log-C_maxBlood_ with a median 37% maximal inhibition compared to baseline CNA measured before administration of the first post-transplant tacrolimus dose. The authors calculated C_maxBlood_ and C_maxPBMC_ producing 50% inhibition of CNA (IC_50_) of 18 μg/L and 100 pg/10^6^ cells, respectively. Similar to an *in vitro* IC_50_ of 160 pg/10^6^ cells calculated using PBMC isolated from healthy volunteers (Tron et al., 2019). Using a population pharmacokinetic model developed in the same study (discussed below in *Whole blood and PBMC tacrolimus pharmacokinetics*), Tron et al. (2020) estimated that in recipients with C_0Blood_ of <4, 4-6 or 6–10 μg/L only 13, 39, and 42%, respectively, were likely to attain C_maxPBMC_ greater than the IC_50_. In comparison, Capron *et al.* reported mean (s.d.) C_0PBMC_ of 90.9 (41.2) *vs* 33.8 (16.7) pg/10^6^ cells in liver transplant patients with no/mild *vs* moderate/severe biopsy graded rejection; and 48.7 (11.9) *vs* 22.0 (6.1) pg/10^6^ cells in patients without *vs* with clinically significant rejection (Capron et al., 2012). This may suggest that *in vivo* prevention of rejection potentially occurs at tacrolimus PBMC concentrations lower than those required for *ex vivo* inhibition of PBMC CNA. Interestingly, Tron *et al.* also reported significant inter-individual variability in baseline CNA (coefficient of variation (CV) = 66%), indicating a considerable component of inter-individual pharmacodynamic variability. Most recently, Fontova et al. (2021) also reported an average 29% maximum inhibition of CNA within a 12 h dosing interval and a significant inverse correlation between AUC_Blood_ and the area under the percentage inhibition of CNA versus time curve in renal transplant recipients. Although they also measured AUC_PBMC_ a similar analysis was not performed.

Han et al. (2016) investigated C_0Blood_ and C_0PBMC_ in 213 stable renal transplant recipients, and quantitated interferon-γ (IFN-γ) and interleukin-2 (IL-2) expressing T-cells in a subset of 39 recipients grouped according to C_0PBMC_. They reported that, following *ex vivo* activation with phorbol-12-myristate 13-acetate and ionomycin, the proportion of CD3^+^CD4^+^IFN-γ^+^, CD3^+^CD4^+^IL-2^+^ and CD3^+^CD8^+^IL-2^+^ T-cells was significantly greater in the low C_0PBMC_ group. However, a significantly higher proportion of CD3^+^CD4^+^IFN-γ^+^ and CD3^+^CD4^+^IL-2^+^ cells was also observed when the comparisons were based on low versus high C_0Blood_.

In general, these studies show that both blood and PBMC tacrolimus concentrations correlate with inhibition of CNA or measures of lymphocyte activation, although the correlations appear stronger using PBMC concentrations. The reports are also consistent with other studies indicating that tacrolimus does not completely inhibit lymphocyte CNA (Fukudo et al., 2005) and support the role of triple maintenance immunosuppression in allowing lower tacrolimus exposures whilst still maintaining a relatively low risk of rejection.

### Whole Blood and PBMC Tacrolimus Pharmacokinetics

Tacrolimus PBMC concentrations have been measured in recipients of liver, kidney and heart transplants, with mean C_0PBMC_ ranging from 22.5–266 pg/10^6^ cells and corresponding mean C_0Blood_ between 3.4–10.5 μg/L (Capron et al., 2010; Capron et al., 2012; Lemaitre et al., 2013; Lemaitre et al., 2015; Han et al., 2016; Klaasen et al., 2018; Romano et al., 2018; Francke et al., 2020; Tron et al., 2020; Fontova et al., 2021) (Supplementary Table S1). Most studies report greater inter-individual variability in PBMC compared to blood tacrolimus concentrations, with CVs for C_0PBMC_ and C_0Blood_ ranging from 40 to 110% and 20–57%, respectively (Capron et al., 2010; Capron et al., 2012; Lemaitre et al., 2013; Lemaitre et al., 2015; Han et al., 2016; Klaasen et al., 2018; Romano et al., 2018; Tron et al., 2020). Whether this reflects greater physiological variability or analytical variability is unclear. Only one study has assessed variability of the PBMC preparation step and reported a CV of 7.3%, which was greater than the intra-assay imprecision but similar to inter-assay imprecision (Klaasen et al., 2018). This provides some confidence that inter-individual variability in tacrolimus PBMC pharmacokinetics may indeed be greater than in blood, supporting a potential benefit of measuring concentrations in PBMC.

In keeping with a greater variability of tacrolimus PBMC pharmacokinetics, a lack of statistically significant correlation between C_0Blood_ and C_0PBMC_ has been reported in some studies (Capron et al., 2010; Capron et al., 2012; Lemaitre et al., 2013; Romano et al., 2018). However, others have found significant, albeit weak, correlations between blood and PBMC tacrolimus concentrations at C_0_ (Pensi et al., 2015; Han et al., 2016; Klaasen et al., 2018; Francke et al., 2020; Tron et al., 2020), 1.5 h post dose (Klaasen et al., 2018) and C_max_ (Tron et al., 2020), and between AUC_Blood_ and AUC_PBMC_ (Lemaitre et al., 2015; Tron et al., 2020; Fontova et al., 2021). Tron et al. (2020) also reported that C_0Blood_ correlated with both C_maxPBMC_ and AUC_PBMC_. Fontova *et al.* recently demonstrated circadian variability in both blood and PBMC tacrolimus pharmacokinetics, with higher blood and PBMC tacrolimus exposures following the morning (compared to evening) dose; and stronger correlations between the 0–12 h tacrolimus AUC in blood or PBMC and the corresponding 12 h (C_12)_, rather than the pre-dose (C_0_) trough concentrations (Fontova et al., 2021). Previous studies have also reported better correlations between C_12Blood_ and AUC_Blood_ (Barraclough et al., 2011; Marquet et al., 2018).

Since T-cells can comprise between 12–92% of total cell numbers in PBMC preparations, one study has compared tacrolimus C_0_ in whole blood, PBMC, purified CD4^+^ T-cells and purified CD19^+^ B-cells of kidney transplant recipients (Romano et al., 2018). In this study, tacrolimus C_0_ were higher in T- and B-cells compared to PBMC, and there was a significant correlation between C_0_ in blood and T-cells, but not between C_0_ values in any of the other matrices. A study in healthy volunteers administered tacrolimus also found a significant correlation between tacrolimus concentrations in blood and T-cells (but not PBMC) (In 't Veld et al., 2019), and in contrast to the study in transplant recipients, tacrolimus concentrations in T-cells were lower than those in PBMC. Taken together, all studies indicate that across liver, renal and heart transplant groups, the relationship between blood and PBMC tacrolimus concentrations is relatively modest and may be affected by the composition of the PBMC fraction. This may, in part, contribute to the poor correlation between blood and PBMC tacrolimus concentrations, as rejection and inflammation are likely to alter the cellular composition of this matrix.

As previously discussed, only unbound tacrolimus distributes from plasma into erythrocytes and the other cells contained in whole blood. Thus, changes in haematocrit, red cell number and plasma proteins may impact the proportion of tacrolimus in whole blood that is distributed within the PBMC fraction. In addition, leucocyte uptake and efflux of tacrolimus may involve carrier mediated processes which may be subject to saturability, induction, inhibition and genetic polymorphisms. All these processes may also affect the rate and extent of tacrolimus distribution within the different compartments in whole blood. The C_0PBMC_/C_0Blood_ ratio is an indication of the proportion of whole blood tacrolimus that is distributed within PBMC. Identifying covariates that determine this ratio may assist in the prediction of tacrolimus C_0PBMC_ from C_0Blood_.

In kidney transplant recipients, Capron et al. (2010) used multiple linear regression to assess pharmacogenetic and other clinical variables (Table 2A) as predictors of C_0PBMC_, C_0PBMC_/dose and C_0PBMC_/C_0Blood_. They reported that recipient *ABCB1* SNPs (1199GA, 3435TT), *CYP3A5* non-expressor genotype, a CYP3A5*3—*ABCB1* 1199GA interaction, and the log of mean corpuscular volume (MCV) were independent determinants of C_0PBMC_/Dose one week after renal transplantation, whilst at steady-state *ABCB1* 1199GA was no longer significant. The effect of *CYP3A5* most likely reflected the TDM guided lower doses of tacrolimus in non-expressors. In contrast, *ABCB1* SNPs (1199GA, 3435CT, 3435TT) and total plasma protein concentrations were independent determinants of C_0PBMC_/C_0Blood_ one week post-transplantation, whilst at steady-state *ABCB1* 3435TT was no longer significant (Table 2A). In this study the variants of *ABCB1* (the gene coding for P-glycoprotien) were independent predictors of a higher C_0PBMC_/C_0Blood_ ratio (Table 2A), consistent with reduced efflux of tacrolimus from PBMC. In addition, high total plasma protein was an independent predictor of a lower ratio (Table 2A), consistent with increased binding of tacrolimus to plasma proteins and therefore less unbound tacrolimus available for distribution into PBMC. Unfortunately, haematocrit does not appear to have been tested as a covariate in this analysis.

**TABLE 2:** Predictors of the ratio of PBMC:blood tacrolimus trough concentrations or 12-h AUCs.

StudyTime post-transplantInteracting drugs	Statistical analysis	Covariates tested	Significant predictors/correlations
A. Kidney Transplants			
Capron et al. (2010)	Multiple linear regression	Recipient Genetics:	Day 7 C_0PBMC_/C_0Blood_:
Day 7 and steady-state (1 month)Not excluded		*ABCB1* 1199G>A, 2677G>T/A, 3435C>TCYP3A5*3Other: age, plasma bilirubin, plasma creatinine, total PPr, MCV	1199GA β = 0.3148 *p* = 0.00033435CT β = 0.1152 *p* = 0.02383435TT β = 0.1727 *p* = 0.0033PPr β = −1.2364 *p* = 0.0051Steady-state C_0PBMC_/C_0Blood_:1199GA β = 0.4123 *p* = 0.0,0883435CT β = 0.1435 *p* = 0.0125PPr β = −0.9867 *p* = 0.0328
Han et al. (2016)Steady-state (up to 14 years post-transplant)Excluded	ANCOVA	Recipient Genetics:*ABCB1* 1236C>T, 2677G>T/A, 3435C>TOther: age; sex; donor type; previous transplantation; diabetes mellitus; delayed graft function; acute rejection; recurrent original disease; CNI-nephrotoxicity; duration of transplantation	Steady-state C_0PBMC_/C_0Blood_: sex F = 5.111 *p* = 0.025haematocrit F = 4.579 *p* = 0.034transplant duration F = 7.233 *p* = 0.008
Francke et al. (2020)Steady-state (3 months)Excluded (up to 3 months post-transplant)	Multiple linear regression	Recipient Genetics:*ABCB1* 1199G>A, 3435C>TCYP3A4*22, CYP3A5*3Other: age, gender, haematocrit, serum albumin, serum creatinine	Steady-state C_0PBMC_/C_0Blood_:age β = 0.0229, *p* = 0.048albumin β = 0.1275, *p* = 0.007haematocrit β = −16.138, *p* < 0.001
B. Liver Transplants			
Tron et al. (2020)Approximately 1 weekExcluded	^1^Mann-Whitney or Kruskal-Wallis tests, with Bonferroni correction, as appropriate^2^Univariate correlation analyses	^1^Donor & Recipient Genetics:*ABCB1* 1199G>A, 1236C>T, 2677G>T/A, 3435C>TCYP3A4*22; CYP3A5*3 ^2^Other: age, sex, body weight, albumin, haematocrit, PBMC cell number	^1^Week one AUC_PBMC_/AUC_Blood_: recipient ABCB1 2677TT (*p* < 0.05)recipient ABCB1 1236/2677/3435 homozygous TTT (*p* < 0.05)^2^Week one AUC_PBMC_/AUC_Blood_: haematocrit r = -0.34, *p* = 0.036

MCV = mean corpuscular volume, PPr = plasma protein. Statistical analysis performed for ^1^pharmacogenetic or ^2^other comparisons in (Tron et al., 2020).

In contrast, in stable renal transplants, Han et al. (2016) found no association between recipient *ABCB1* SNPs and C_0PBMC_/C_0Blood_, but did find a significant association with sex, haematocrit and transplant duration using analysis of covariance (Table 2A). In addition, haematocrit and transplant duration were also significantly associated with C_0PBMC_. Unfortunately, tacrolimus dose was not investigated. More recently, using multiple linear regression in kidney transplants, Francke et al. (2020) also found no effect of recipient *ABCB1* 1199/3435 variant alleles, CYP3A5*3 or CYP3A4*22 on C_0PBMC_/C_0Blood_ ratio but age, albumin and haematocrit were independent predictors of the ratio at 3 months post-transplant (Table 2A), similar to (Han et al., 2016).

Tron et al. (2020) developed a 2-compartment population pharmacokinetic model describing the relationship between blood and PBMC tacrolimus concentrations approximately 1 week after liver transplantation. Although they did not find any demographic or pharmacogenetic covariates that significantly improved their model, univariate analyses of model-derived AUCs and observed C_0_ and C_max_ revealed a significant but weak inverse correlation between haematocrit and AUC_PBMC_ and between haematocrit and the ratio of AUC_PBMC_/AUC_Blood_ (Table 2B), similar to the studies in renal transplants. High haematocrit is consistent with a larger erythrocyte binding compartment and therefore less unbound tacrolimus available for distribution into PBMC. In the above renal and hepatic transplant studies, haematocrit was the only consistent determinant of the proportion of whole blood tacrolimus that is present within PBMC. Haematocrit is also a significant covariate in many solid organ transplant population models of whole blood tacrolimus pharmacokinetics (Brooks et al., 2016).

Tron *et al.* also found that recipient (but not donor) 2677TT and 1236/2677/3435 homozygous triple variant *ABCB1* SNPs were associated with a lower AUC_PBMC_/AUC_Blood_ ratio, whilst the recipient *ABCB1* 1199A variant allele had no effect (Table 2B) (Tron et al., 2020). In this study the lower AUC_PBMC_/AUC_Blood_ ratio in carriers of the *ABCB1* 3435T variant contradicts its association with higher C_0PBMC_/C_0Blood_ ratios reported by Capron et al. (2010) 1 week after renal transplantation. In addition, the association between recipient *ABCB1* 1199A variant and C_0PBMC_/C_0Blood_ ratios reported by Capron et al. (2010) 1 week after renal transplantation was not observed for the AUC ratio reported by Tron et al. (2020). In kidney transplants, recipient genotypes would relate to hepatic, intestinal and PBMC enzyme/transporter activities (Capron et al., 2010), whilst in liver transplants (Tron et al., 2020) recipient genotypes would relate to intestinal (not hepatic) and PBMC activities, potentially explaining some of the pharmacogenetic discordance between studies. Additionally, relatively small sample sizes and very small numbers of patients who were carriers of variant alleles may also have contributed to discordant observations.

Although *ABCB1* genetic polymorphisms are not major determinants of tacrolimus blood clearance, the *ABCB1* 1199G>A SNP has been shown to increase *in vitro* intra-cellular accumulation of tacrolimus in HEK293 and K562 recombinant cell lines (Dessilly et al., 2014). However, its relative role in the net efflux of tacrolimus from PBMC is unknown and currently its effects on the PBMC:blood concentration ratio is contradictory. Unfortunately, direct comparison of these studies is difficult due to differences in the preparation of PBMC; cellular composition of PBMC, sample size; ethnicity of transplant recipients; exclusion of drugs that interact with CYP3A and P-glycoprotein; transplant duration; covariates investigated; statistical analyses and corrections for multiple comparisons.

Most of the above studies have addressed inter-individual variability in the C_0PBMC_/C_0Blood_ ratio but not intra-individual variability. Han et al. (2016) measured C_0PBMC_/C_0Blood_ on two occasions in a small subset of renal transplant recipients and reported that the ratio measured >1 year post-transplant was significantly lower than that measured in the first year. In contrast, two later studies with larger sample sizes found no effect of time post-transplant on C_0PBMC_/C_0Blood_ when subjects were repeatedly sampled at different time-points after transplantation (Klaasen et al., 2018; Francke et al., 2020). They reported median or mean intra-individual CVs of 45% (range 5.9–88%) (Klaasen et al., 2018) and 39.0% (range 3.5–173.2%) (Francke et al., 2020), which were lower than the CVs for inter-individual variability (Francke et al., 2020). Interestingly, in the patients with the greatest intra-individual variability, the variability could not be explained by changes in haematocrit (Francke et al., 2020).

One important determinant of tacrolimus distribution (and activity), which has not been addressed, is intra-cellular binding capacity (Figure 1). FKBP12 is the major erythrocyte cytoplasmic protein to which tacrolimus binds (Nagase et al., 1994). Whilst haematocrit is an estimate erythrocyte numbers, it does not address variability in erythrocyte expression of FKBP12. Although inhibition of calcineurin in lymphocytes is mediated by the tacrolimus-FKBP12 complex, tacrolimus also binds to other FKBPs whose expression differs between tissues and cell types (Baughman et al., 1997). Thus, differences or changes in the expression of FKBPs are likely to affect both inter- and intra-individual variability in whole blood and PBMC tacrolimus pharmacokinetics. In addition, variability in FKBP expression within lymphocytes may also affect the degree of calcineurin inhibition by tacrolimus (Kung and Halloran, 2000).

## Allograft Tacrolimus Concentrations

### Rejection

In 1992 Sandborn *et al.* reported that liver transplant recipients with cellular rejection had lower graft tissue ciclosporin concentrations than those without (Sandborn et al., 1992). They later expanded these observations to tacrolimus in a group of 17 *de novo* liver transplant recipients administered maintenance immunosuppression consisting of tacrolimus and prednisolone (Sandborn et al., 1995). Seven of the 17 patients developed nine episodes of histological rejection and had graft tacrolimus concentrations (measured using clinical biopsy samples) that were significantly lower than those in the patients without rejection. However, they found no difference in plasma tacrolimus concentrations between patients with and without rejection. These early reports (Table 1A) provided much of the impetus for better understanding the role of graft tacrolimus concentrations in determining risk of rejection. In these early studies both plasma and tissue tacrolimus concentrations were measured by an immunoassay with significant cross-reactivity to tacrolimus metabolites (Wallemacq et al., 2009). Since the metabolite/parent tacrolimus concentration ratio changes over a dosing interval and may also differ between plasma and other tissues, these early observations may have been subject to significant analytical bias. Capron *et al.* next investigated the relationship between graft tacrolimus concentrations and rejection in 146 *de novo* liver transplant recipients administered tacrolimus and corticosteroid maintenance immunosuppression (Table 1A) (Capron et al., 2007). They reported that day 7 graft tacrolimus concentrations (measured by a specific LC-MS/MS method) were significantly lower in patients with moderate/severe histological rejection compared to those with no (or mild) rejection, and that there was a strong first-order exponential correlation between Banff histology score and hepatic tacrolimus concentrations (r^2^ = 0.98 *p* = 0.002). A cut-off hepatic tacrolimus concentration of 30 pg/mg of tissue predicted clinically significant rejection with 89% sensitivity and 98% specificity. In comparison, there was no difference in C_0Blood_ in patients with or without mild/moderate rejection. However, C_0Blood_ were measured with an immunoassay also associated with significant metabolite cross-reactivity (Wallemacq et al., 2009). This was followed by another study (also discussed in *Rejection and nephrotoxicity*) in liver transplant recipients (Table 1A) again reporting significantly lower liver tacrolimus concentrations (and C_0PBMC_) in patients with moderate/severe histological rejection compared to those with no/mild rejection, and a significant relationship between liver tacrolimus concentrations (and C_0PBMC_) and Banff scores (Capron et al., 2012). Even though C_0Blood_ were measured by a relatively specific immunoassay there was still no association between C_0Blood_ and rejection.

Two studies (Table 1A) have recently investigated potential relationships between renal graft tacrolimus concentrations and clinical outcomes (Zhang et al., 2020; Sallustio et al., 2021). In 52 renal transplant recipients there was no difference in renal tacrolimus concentrations between patients with or without histologically classified subclinical acute rejection at 3 months or 1 year post transplantation (Zhang et al., 2020). In a larger study of 132 renal transplant recipients, biopsy-proven rejection was similarly not associated with renal tacrolimus concentrations (Sallustio et al., 2021). In both studies, patients received triple maintenance immunosuppression with tacrolimus, mycophenolic acid and prednisolone and rejection episodes were observed in 21% of patients (Zhang et al., 2020) and 44% of biopsy samples (Sallustio et al., 2021). These later studies contrast with the earlier results in hepatic transplantation. However, the renal transplant recipients were administered current triple maintenance immunosuppression, whereas the hepatic transplant recipients were administered maintenance immunosuppression of tacrolimus monotherapy or tacrolimus and a steroid (Table 1A). Therefore, the utility of hepatic tacrolimus concentrations as predictors of rejection with modern triple therapy is yet to be determined.

### Nephrotoxicity

Despite a reduction in the target C_0Blood_ therapeutic range, evidence of chronic tacrolimus induced nephrotoxicity is still present in 34 and 72% of renal allograft biopsies by 5 and 10 years post-transplantation (Nankivell et al., 2016). Although high C_0Blood_ are associated with increased risk of nephrotoxicity, it is unclear whether renal CNI concentrations may be better predictors. Of the two studies that have measured intra-renal tacrolimus concentrations in clinical allograft biopsies (Table 1A), only one has investigated nephrotoxicity, reporting that the relationship between blood and renal tacrolimus concentrations may be different (steeper) in patients with acute nephrotoxicity compared to those without (Sallustio et al., 2021). However, these results were based on a very small incidence of acute nephrotoxicity and require confirmation.

### Whole Blood and Allograft Tacrolimus Pharmacokinetics

Measurement of allograft tacrolimus concentrations is ethically limited to the use of biopsies collected for clinical assessment of graft dysfunction or as part of established routine clinical monitoring protocols. Thus, there are no clinical data on graft tacrolimus AUCs. However, similar to the studies of PBMC tacrolimus exposures, Capron *et al.* found no correlation between C_0Blood_ and graft tacrolimus concentrations in liver transplant recipients, using a relatively non-specific immunoassay to measure C_0Blood_ (Capron et al., 2007) and also in a later publication in which C_0Blood_ were measured with a more specific immunoassay (Capron et al., 2012). In contrast, the latter study reported a good correlation (r^2^ = 0.55, *p* = 0.001) between C_0PBMC_ and hepatic tacrolimus concentrations (Capron et al., 2012), possibly indicating that both PBMC and hepatic tacrolimus concentrations are more closely related to unbound plasma tacrolimus concentrations than those in whole blood. In renal transplant recipients, weak correlations have been reported between C_0Blood_ and graft tacrolimus concentrations with r^2^ values of 0.13 (*p* = 0.01) (Zhang et al., 2020) and 0.19 (*p* = 7.4 × 10^−10^) (Sallustio et al., 2021). In addition, a better correlation between dose and renal tacrolimus concentrations than between dose and C_0Blood_ (Sallustio et al., 2021) has also been reported, again potentially indicating that renal tacrolimus concentrations better reflect unbound plasma tacrolimus concentrations, hence, dose.

Similar to PBMC, there appears to be greater inter-individual variability in intra-graft tacrolimus concentrations than C_0Blood_ (Supplementary Table S1), with mean (s.d.) concentrations of 91.3 (52.2) pg/mg of tissue and 8.9 (3.0) μg/L, respectively, and CVs of 57 and 34%, respectively in liver transplant recipients (Capron et al., 2012). In renal transplantation, C_0Blood_ and graft tacrolimus concentrations ranged from 2.6 to 52.3 μg/L and 33–828 pg/mg of tissue, respectively (Sallustio et al., 2021). Analytical variability in measurement of intra-renal tacrolimus concentrations appears relatively small, with one study reporting intra- and inter-assay CVs between 5.9 and 14.1% for replicate analyses of *in vivo* renal cortical tissue tacrolimus concentrations (Noll et al., 2013).

Only one group (Elens et al., 2007) has investigated the effect of *ABCB1* and *CYP3A5* genetic polymorphisms on hepatic tacrolimus concentrations in liver transplantation (post hoc analysis of (Capron et al., 2007) in Table 1A). Using multiple linear regression analysis, donor *ABCB1* 2677 G/T, T/T and G/A, 1199G/A and day 7 log plasma bilirubin concentrations were independent predictors of day 7 hepatic tacrolimus concentrations, whist the same genotypes (but not bilirubin) were independent predictors of dose-corrected hepatic tacrolimus concentrations (Elens et al., 2007). In renal transplants, donor or recipient *ABCB1* and *CYP3A5* genetic polymorphisms had no effect on renal tacrolimus concentrations (Zhang et al., 2020; Sallustio et al., 2021), but C_0Blood_ (p = 1 × 10^−8^), dose (*p* = 0.02) and acute nephrotoxicity (main effect *p* = 0.01 and first-order interaction with C_0Blood_ *p* = 0.002) were independent predictors of renal tacrolimus concentrations (Sallustio et al., 2021). Interestingly, a greater role of P-glycoprotein in determining hepatic *versus* renal tacrolimus concentrations is supported by animal work showing that knockout of P-glycoprotein expression in mice results in increased tissue/blood tacrolimus concentration ratios in liver but not in kidneys (Yokogawa et al., 1999).

## Conclusion

Although investigation of PBMC and allograft tissue as alternate matrices for tacrolimus TDM has been conducted for more than 20 years, there is little consistent evidence for a clinical benefit with respect to the prediction of rejection. Most studies are limited by their retrospective or post-hoc design, small sample sizes and insufficient statistical power. The only evidence of a strong predictive value of PBMC and allograft tacrolimus C_0_ with respect to rejection was in liver transplant recipients administered immunosuppression regimens that are no longer used clinically (Capron et al., 2012). The results have not been independently replicated in liver or other transplant groups using current immunosuppressant regimens. Only one study has reported an association between renal tacrolimus concentrations and histological evidence of acute nephrotoxicity (Sallustio et al., 2021). Thus, well-designed and powered prospective clinical studies are still required to determine whether TDM of tacrolimus using PBMC or graft concentrations offers a significant clinical benefit compared to current TDM based on blood tacrolimus concentrations. Harmonisation of analytical methods may be an important initial step to significantly facilitate comparisons between laboratories and generalisation of results (Lemaitre et al., 2020).

Population pharmacokinetic modelling could provide a robust sparse sampling strategy with which to investigate the relationship between tacrolimus concentrations in whole blood and PBMC (or allograft tissue) and pharmacodynamics (e.g., rejection), and could also allow for the assessment significant covariates, including the effects of drugs that may interfere with tacrolimus distribution (e.g., inhibitors/inducers of efflux or uptake proteins such as P-glycoprotein or SLCO1B proteins (Elens et al., 2007; Boivin et al., 2013)). Validated models may facilitate prediction of PBMC or allograft tacrolimus concentrations without the need to carry our frequent actual analysis in patients. However, like whole blood, PBMC are a heterogenous collection of cells and their use may have limitations similar to the use of whole blood.
