# Supplementary material for: Monitoring Intra-cellular Tacrolimus Concentrations in Solid Organ Transplantation: Use of Peripheral Blood Mononuclear Cells and Graft Biopsy Tissue
Source: Front Pharmacol. 2021 Oct 26;12:733285. doi: 10.3389/fphar.2021.733285 (PMC8576179; doi:10.3389/fphar.2021.733285)
Supplement: Supplementary file 1 [file Table1.docx]

**Supplementary Table.** Comparative tacrolimus concentration data reported in plasma, blood, PBMC and allograft tissue

| **Study** | **Time post-transplant** | **Blood [Tac] (ng/mL)^1^** | **PBMC [Tac] (pg/10^6^ cells)^1^** | **Allograft [Tac] (pg/mg)^1^** |
| --- | --- | --- | --- | --- |
| A. Liver Transplants | | | | |
| Sandborn et al. 1995 (n=17) | Up to 8 weeks | Range 0.2-2.3 (plasma) | ND | Range 0-624 |
| Capron et al. 2007 (n=146) | Day 7 | C_0_: 7.3 (2.7)^2^ | ND | 42.9 (57.2) |
| Elens et al. 2007 (n=146) | Day 1  Day 7 | C_0_: 1.5-4.6^3^ | ND | 16.7-99.3^3^ |
| Capron et al. 2012 (n=90) | Day 7 | C_0_: 8.9 (3.0) | C_0_: 65.4 (41.8) | C_0_: 91.3 (52.2) |
| Lematire et al. 2015 (n=10) | Day 1  Day 7 | C_max_: 13.0 (9.6) C_12_: 6.9 (3.1) AUC: 112 (66)^5^  C_max_: 8.8 (5.0) C_12_: 5.4 (3.1) AUC: 81 (37)^5^ | C_max_: 117 (115) C_12_: 71.3 (78.5) AUC: 909 (904)^5^  C_max_: 68 (49) C_12_: 39.5 (38.8) AUC: 673 (602)^5^ | ND |
| Rayar et al. 2018 (n=41) | Days 1-7 | Data only presented graphically. | Data only presented graphically. | ND |
| Tron et al. 2020 (n=32) | SS Day 7-10 | C_0_: 6.4 (2.2) C_max_: 16.4 (6.9) AUC: 108.9 (38.9)^5^ | C_0_: 37.2 (17.7) C_max_: 78.1 (37.1) AUC: 491.6 (223.0–1127.2)^5,7^ | ND |
| B. Kidney Transplants | | | | |
| Capron et al. 2010 (n=96) | Day 7  SS (1 month) | C_0_: 13.2 (12.4–14.0)^4^  C_0_:11.2 (10.6–11.7)^4^ | C_0_: 71.6 (19.4)  C_0_: 76.6 (19.9) | ND |
| Han et al. 2016 (n=214) | SS ≤ 1 year 1-2 years 2-4 “ 4-6 “ 6-10 “ >10 “ | C_0_: 6.8 (2.1) C_0_: 5.5 (1.1) C_0_: 4.7 (1.7) C_0_: 4.4 (1.5) C_0_: 3.8 (1.5) C_0_: 3.4 (1.5) | C_0_: 75.2 (33.3) C_0_: 61.2 (25.3) C_0_: 43.3 (36.8) C_0_: 42.7 (25.8) C_0_: 31.4 (16.7) C_0_: 29.2 (13.9) | ND |
| Klaasen et al. 2018 (n=29) | 1 week  6 weeks  1 year | C_0_: 5.0 (4.5–6.2)^6^ C_1.5_: 10.5 (7.1–16.4)^6^  C_0_: 6.0 (5.7–6.8)^6^ C_1.5_: 8.3 (7.0–10.7)^6^  C_0_: 5.4 (4.0–6.6)^6^ C_1.5_: 9.1 (7.1–11.5)^6^ | C_0_: 22.5 (21.0–35.2)^6^ C_1.5_: 43.9 (36.3–61.8)^6^  C_0_: 33.0 (22.9–41.3)^6^ C_1.5_: 29.9 (25.3–55.2)^6^  C_0_: 27.4 (25.1–36.3)^6^ C_1.5_: 27.2 (21.0–45.1)^6^ | ND |
| Romano et al. 2018 (n=20) | SS (time unclear) | 5.0 (2.7) | 266 (153) | ND |
| Francke et al. 2020 (n=175) | 3 months  6 months  12 months | C_0_: 7.5 (6.1–9.2)^6^  C_0_: 6.6 (5.6–8.2)^6^  C_0_: 5.9 (4.3–7.7)^6^ | C_0_: 26.5 (18.8–37.0)^6^  C_0_: 23.8 (16.5–33.4)^6^  C_0_: 20.8 (12.5–31.4)^6^ | ND |
| Zhang et al. 2020 (n=52) | 3 months  1 year | C_0_: 5.2 (1.3)  C_0_: 5.2 (1.2) | ND | Data only presented graphically. |
| Sallustio et al. 2021 (n=132) | SS 15 (8-80)^7^ days | C_0_: 10.8 (5.2) | ND | 211 (111) |
| Fontova et al. 2021 (n=25) | SS >6 months  a.m. dose  p.m. dose | C_max_: 18.2 (15.6–21.3)^4^ C_12_: 6.7 (6.0–7.5)^4^ AUC: 115.4 (104.2-127.9)^4,5^  C_max_: 11.1 (9.4–13.2)^4^ C_24_: 6.0 (5.3–6.8)^4^ AUC: 92.4 (81.5–104.8)^4,5^ | C_max_: 95.0 (72.0–125.3)^4^ C_12_: 32.1 (25.4–40.6)^4^ AUC: 601.4 (478.1–756.5)^4,5^  C_max_: 61.9 (47.6–80.5)^4^ C_24_: 28.5 (22.4–36.2)^4^ AUC: 477.3 (373.7–609.7)^4,5^ | ND |
| C. Heart Transplants | | | | |
| Lemaitre et al. 2013 (n=24) | Not stated | C_0_: 8.1 (2.6) | C_0_: 40.0 (29.0) | ND |

^1^Presented as mean (s.d.) unless otherwise indicated; ^2^Over days 5-7; ^3^Range of geometric means; ^4^Geometric mean (95% confidence intervals); ^5^ng.hr/mL or pg.hr/10^6^ cells; ^6^median (interquartile range); ^7^median (range). Abbreviations: Tac = tacrolimus, [Tac] = tacrolimus concentration, SS = steady state, ND = not determined.
